# Genomic Heterogeneity of Methicillin Resistant *Staphylococcus aureus* Associated with Variation in Severity of Illness among Children with Acute Hematogenous Osteomyelitis

**DOI:** 10.1371/journal.pone.0130415

**Published:** 2015-06-18

**Authors:** Claudia Gaviria-Agudelo, Chukwuemika Aroh, Naureen Tareen, Edward K. Wakeland, MinSoo Kim, Lawson A. Copley

**Affiliations:** 1 Department of Pediatric Infectious Disease, University of Texas Southwestern Medical Center, Dallas, Texas, United States of America; 2 Department of Immunology, University of Texas Southwestern Medical Center, Dallas, Texas, United States of America; 3 Children’s Medical Center, Dallas, Texas, United States of America; 4 Texas Scottish Rite Hospital for Children, Dallas, Texas, United States of America; 5 Department of Orthopaedic Surgery, University of Texas Southwestern Medical Center, Dallas, Texas, United States of America; 6 Department of Biomedical Informatics, University of Texas Southwestern Medical Center, Dallas, TX, United States of America; University Hospital Münster, GERMANY

## Abstract

**Introduction:**

The association between severity of illness of children with osteomyelitis caused by Methicillin-resistant *Staphylococcus aureus* (MRSA) and genomic variation of the causative organism has not been previously investigated. The purpose of this study is to assess genomic heterogeneity among MRSA isolates from children with osteomyelitis who have diverse severity of illness.

**Materials and Methods:**

Children with osteomyelitis were prospectively studied between 2010 and 2011. Severity of illness of the affected children was determined from clinical and laboratory parameters. MRSA isolates were analyzed with next generation sequencing (NGS) and optical mapping. Sequence data was used for multi-locus sequence typing (MLST), phylogenetic analysis by maximum likelihood (PAML), and identification of virulence genes and single nucleotide polymorphisms (SNP) relative to reference strains.

**Results:**

The twelve children studied demonstrated severity of illness scores ranging from 0 (mild) to 9 (severe). All isolates were USA300, ST 8, SCC mec IVa MRSA by MLST. The isolates differed from reference strains by 2 insertions (40 Kb each) and 2 deletions (10 and 25 Kb) but had no rearrangements or copy number variations. There was a higher occurrence of virulence genes among study isolates when compared to the reference strains (p = 0.0124). There were an average of 11 nonsynonymous SNPs per strain. PAML demonstrated heterogeneity of study isolates from each other and from the reference strains.

**Discussion:**

Genomic heterogeneity exists among MRSA isolates causing osteomyelitis among children in a single community. These variations may play a role in the pathogenesis of variation in clinical severity among these children.

## Introduction

Within the past decade there has been an increase in the incidence of community acquired methicillin-resistant *Staphyloccocus aureus* (MRSA) as the cause of deep infections in children [1–6]. Severe forms of infection have been associated with disseminated disease, deep venous thrombosis, septic pulmonary emboli, abscess formation requiring surgical drainage, and multifocal skeletal involvement [7–9]. However, some children develop a mild clinical illness which resolves quickly following brief hospitalization and antibiotic treatment without surgery [10]. The pathogenetic mechanisms leading to such variation in clinical manifestations of acute hematogenous osteomyelitis have yet to be clearly established.

It is possible that virulence genes, in isolation or combination, may play a role in the pathogenic behavior of specific strains, but this has been difficult to prove with traditional approaches to bacterial genetics using pulse field gel electrophoresis (PFGE) or multi-locus sequence typing (MLST) because they focus on macromolecular patterns or on core housekeeping genes which are common to all *Staphylococcus aureus* strains [11,12]. Bacterial isolates from persons with nasal carriage, community-acquired invasive disease, and hospital-acquired invasive disease appear to be evenly distributed among *S*. *aureus* clonal complexes, suggesting no significant differences in their propensity to cause invasive disease [11]. Research using polymerase chain reaction (PCR) technology to identify the occurrence of individual virulence factors in bacteria isolated from children with severe disease has not led to definitive conclusions due to the complexity and redundancy of the *S*. *aureus* genome [13–15].

After technologies emerged to permit rapid whole genome sequencing of clinical isolates [16–20] several reference strains of *S*. *aureus* were sequenced to provide details of the specific location and composition of virulence genes [16,17,19]. Next-generation sequencing (NGS) permits the identification of single nucleotide polymorphisms (SNPs) and indels (insertions or deletions) with respect to the sequences of reference isolates [21,22]. The purpose of this study is to utilize NGS to evaluate the genomic heterogeneity of MRSA isolates which have been procured from children with osteomyelitis who have varying severity of illness.

## Materials and Methods

### Ethics Statement

This study was conducted according to the principles expressed in the Declaration of Helsinki. The study was approved by the Institutional Review Boards of the University of Texas Southwestern Medical Center and Children's Medical Center of Dallas (IRB #0802–447) and Baylor Institute of Immunology Research (BIIR, IRB # 002–141). Written Informed consent was obtained from the families or legal guardians of all children enrolled in the study. Written Informed Assent was obtained from patients 10 years of age and older prior to any study-related procedure. The study was IRB approved.

Previously healthy children between the ages of birth and 18 years who were admitted to the hospital with acute hematogenous osteomyelitis due to MRSA were consecutively enrolled and prospectively studied after institutional review board (IRB) approval. Acute osteomyelitis was defined as an infection involving bone diagnosed within 2 weeks of the onset of symptoms. The infection was acquired by hematogenous dissemination as opposed to direct inoculation of the bone due to trauma or surgery. All infections were confirmed by the isolation of MRSA obtained from the site of infection under sterile conditions in the operating room. The surgical specimen was plated in the microbiology lab on sheep blood agar and incubated at 37°C. Following culture confirmation of MRSA, the bacterial isolates were catalogued and stored frozen at -80°C in the Children’s Medical Center microbiology laboratory for subsequent processing. Children were excluded from the investigation if they had any underlying medical disorder which may lead to immune compromise such as: congenital immune deficiency, leukemia, transplant, or treatment with chemotherapy or immune-modulatory agents. Also excluded were children with infection due to any bacterial organism other than MRSA.

### Demographic and Clinical Data

The following clinical and laboratory data were gathered: age; gender; ethnicity; infection site; number of surgeries; and total length of hospitalization (including pediatric intensive care unit stay and readmission days). According to our previously described protocol, a severity of illness score [23] was calculated for each child based on the following clinical and laboratory parameters: C-reactive protein (CRP) values at admission, 48 hours, and 96 hours; febrile days on antibiotics; admission respiratory rate (age adjusted); and evidence of disseminated disease (multi-focal involvement, deep venous thrombosis, pulmonary involvement, meningitis, or endocarditis) [23].

### Specimen Processing

Frozen MRSA specimens were thawed and inoculated onto blood-agar plates at 37°C for 8 hours. Isolated colonies were transferred to enriched Mueller Hinton broth media overnight. DNA extraction was performed with DNeasy blood and tissue kits with lysostaphin replacing lysozyme during the initial lysis. (Qiagen, Germantown, MD).

### Microbial Genome Sequencing

2–5 μg of isolated genomic DNA, quantified by Picogreen, was submitted to the UT Southwestern Genomics Core (http://genomics.swmed.edu/) for sequence analysis. Sequencing libraries were produced using TruSeq adaptors (Illumina, Inc., San Diego, CA) with inserts of 300–500 bp in length. DNA quality was analyzed with a Bioanalyzer (Agilent technologies). Sequencing was performed on an Illumina HiSeq 2000 using a paired-end protocol in which 100 bp sequences were obtained from each end of the sequencing library fragments. An average of 6.9 million reads were obtained for the each sample in this study, resulting in an average of 243-fold coverage for the MRSA genome. Sequencing protocols and basic information concerning the sequence generation are available from the UT Southwestern Genomics Core website.

### Optical Mapping

Selected samples representative of the full spectrum of clinical severity of illness scores in our cohort were sent for optical mapping through Opgen’s Mapit service (Opgen Inc., Gaithersburg, MD). The resulting data was analyzed on Opgen’s MapSolver software version 3.2 (Opgen Inc., Gaithersburg, MD). Whole genome maps of each sample, obtained de novo, were aligned against in silico reference strains in order to identify insertion, deletion, re-arrangement, or copy number variation events.

### Sequence Assembly and Analysis

All sequencing data were imported and analyzed using CLC Genomics Workbench (Aarhus, Denmark). Sequences were initially assembled to USA300 FPR3757 and USA300 TCH1516 reference genomes obtained from GenBank, National Center for Biotechnology Informations (http://www.ncbi.nlm.nih.gov/nucleotide). FPR 3757 is a community acquired strain from the wrist abscess of a 36 year old male HIV positive, intravenous drug user in California. TCH 1516 (aka USA 300-HOU-MR) is a community acquired strain obtained from an adolescent female with “severe sepsis syndrome” in Houston. Multi Locus Sequence Typing (MLST) was performed using consensus sequences from each sample for seven housekeeping genes (arc, aroE, glp, gmk, pta, tpi, yqiL) using standard programs available at (saureus.mlst.net) to assign allele and sequence types (STs). Similarly, all samples were analyzed for six virulence genes (alt, essC, geh, hlgA, htrA, and sdrC) as previously proposed for MLST [12]. Analysis of the virulence gene alleles in the study samples was performed via neighbor joining analysis with 10,000 bootstrap (the CLC bio Rapid Neighbor-Joining plugin). Single nucleotide variation and other genetic variation present in the samples were detected using the variant detection function on the genomic workbench using both USA300 strains as reference. Additionally, in some samples which showed large deletions with whole genome mapping, we were able to visually confirm the deletions with the sequence data either in the “stand-alone mapping” or the “tracks” format.

### Phylogenetic Analysis

USA 300 *S*. *aureus* was selected as the clonal complex with the closest phylogenetic similarity to our isolates to permit meaningful comparison with the study strains. In addition to the USA 300 MRSA reference strains (FPR 3757 and TCH 1516), TCH 959 (aka USA 300-HOU-MS) was chosen as a community acquired strain of USA 300 Methicillin-sensitive *Staphylococcus aureus* (MSSA) which was isolated from a buttock abscess of a 12 year old white female. Using MUMmer (http://www.mummer.sourceforge.net/) the reference genome sequences and SNP calling was performed. The haplotypes for each of the study samples were generated with confident homyzygous single nucleotide variant (SNV) sites for which the read fractions supporting major alleles were greater than 0.9 for every sample. These haplotypes were used as the nucleotide input for MEGA 6.06 (Molecular Evolutionary Genetics Analysis) http://www.megasoftware.net/mega.php during the phylogenetic analysis. Maximum Likelihood (ML) phylogenetic reconstruction was peformed with the Jukes-Cantor model using the ML Heuristic Method of Nearest-Neighbor-Interchange (NNI). The initial tree was assembled using maximum parsimony to estimate the evolutionary distances between sequences by computing the proportion of nucleotide differences between each pair of sequences.

### Statistical Analysis

A review of literature [12,13,15–19,24–31] identified 78 genes with virulence potential in the USA300 genome. The number of pathogenic genes in clinical isolates was compared to that of the reference strains using the Mann-Whitney U test. The test was conducted using R (http://www.r-project.org/).

## Results and Discussion

### Clinical Severity of Illness of Affected Children

Twelve children with osteomyelitis due to MRSA were evaluated for clinical severity of illness. Their clinical characteristics are shown in Table 1. All children in this study were culture positive for MRSA which was grown from material obtained directly from the site of infection. No other organisms grew within the cultures of these children. Clinical Severity of Illness Scores (SIS) [23] ranged from 0 to 9 with an average score of 6.1 and median score of 7. The scores were calculated from clinical and laboratory parameters identified by hospital day 5 in all children studied (Table 2). One out of four (25%) children with severity of illness score of 5 or less had a positive blood culture, whereas all eight children (100%) with severity of illness scores greater than 5 had positive blood cultures.

**Table 1:** Clinical and Laboratory Features Determining Severity of Illness Score of Children with Acute Hematogenous Osteomyelitis.

Subject Number	Gender	Age	Location	Bacteremia	CRP initial	CRP 48 hrs	CRP 96 hrs	RR % of mid-range	Febrile days on Abx	ICU Adm	Dissem Disease	Severity of Illness
1	M	13 yr	femur	N	15	5.1	1.7	142%	0	N	N	3
2	M	12 yr	femur	Y	34.7	24.2	30.7	92%	4	Y	Y	9
3	M	12 mo	pelvis	Y	31.4	18.4	20.4	100%	3	N	Y	8
4	F	12 yr	fibula	N	26.4	26.8	7.1	83%	1	N	N	5
5	M	14 mo	humerus	Y	22	18.3	12.4	83%	3	N	Y	8
6	M	19 mo	femur	Y	18.2	20.5	16.1	125%	1	N	N	7
7	M	5 yr	tibia	N	0.1	0.1	2.6	79%	0	N	N	0
8	M	3 yr	tibia	Y	17.7	21.7	27.1	88%	4	Y	Y	9
9	M	8 yr	tibia	Y	23.9	13.4	9.1	125%	1	N	N	6
10	M	4 yr	tibia	Y	16.1	9.3	3.2	100%	0	N	N	3
11	F	12 yr	tibia	Y	55.6	39.6	11.5	171%	3	N	N	8
12	M	2 yr	tibia	Y	22.6	12.1	10.8	100%	3	N	N	7

Male (M); Female (F); C-reactive protein (CRP) initial, 48 hours and 96 hours (mg/dL); Respiratory rate (RR%) – percentage of the mid-range norm breaths per minute for age; Antibiotics (abx); Yes (Y); No (N); Intensive Care Unit (ICU) admission (Adm); Disseminated disease (Dissem Dis) includes: multi-focal infection, pneumonia, septic pulmonary embolism, deep venous thrombosis, septic shock, meningitis, or endocarditis.

**Table 2:** Severity of Illness Scoring Method.

CRP initial:	Respiratory rate:	**Respiratory rate mid-range**	
<10 = 0	≥ 125% of mid range norm = 1	Infant (birth-1yr)	45
10–15 = 1	< 125% = 0	Toddler (1–3yr)	32
>15 = 2		Preschool (3–6yr)	28
	Febrile days on Abx	School-age (6–12yr)	24
CRP 48 hours:	< 2 = 0	Adolescent (12–18yr)	14
<5 = 0	≥ 2 = 1		
5–10 = 1			
>10 = 2	ICU admission:		
	No = 0		
CRP 96 hours:	Yes = 1		
<5 = 0			
5–10 = 1	Disseminated disease:		
>10 = 2	No = 0		
	Yes = 1		

C-reactive protein (CRP); CRP initial, 48 hours and 96 hours unit of measurement – mg/dL; normal (norm); antibiotics (Abx); intensive care unit (ICU); respiratory rate unit of measurement – breaths per minute.

### Bacterial Genetic Heterogeneity

**Fig 1:**
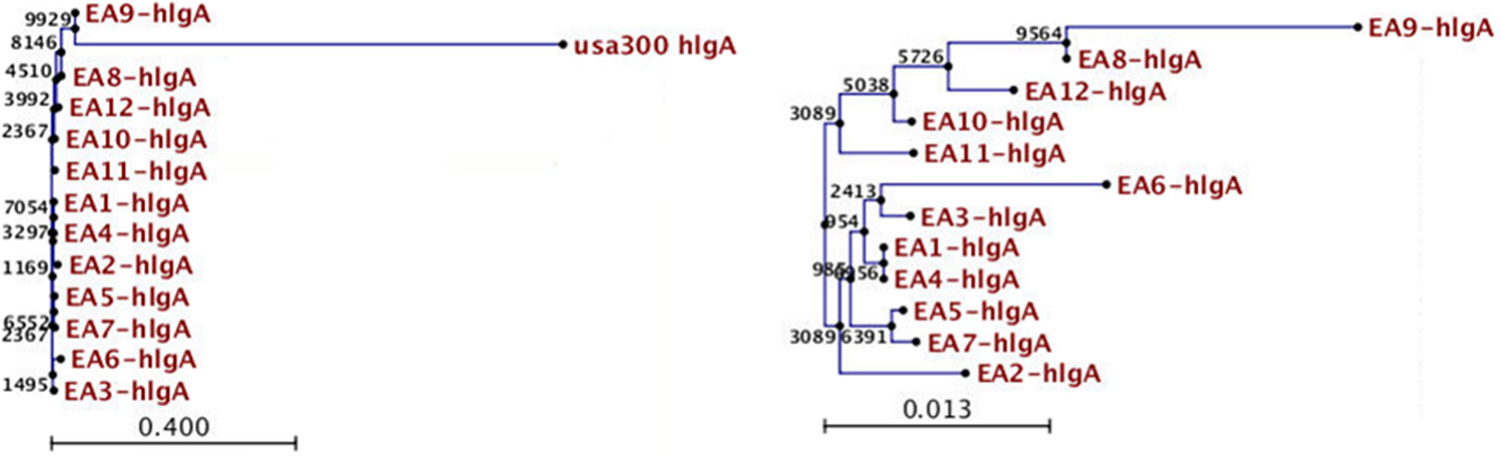
Dendrogram of the differences between study isolates (EA1 through EA12) and the USA 300 reference strain at the gamma hemolysin subunit (HlgA) (left) and dendrogram of the polymorphisms of the gamma hemolysin subunit of the study isolates (right). The tree was generated by neighbor joining analysis with a bootstrap of 10,000. The numbers refer to the confidence of related associations. Higher numbers indicate greater confidence.

**Fig 2:**
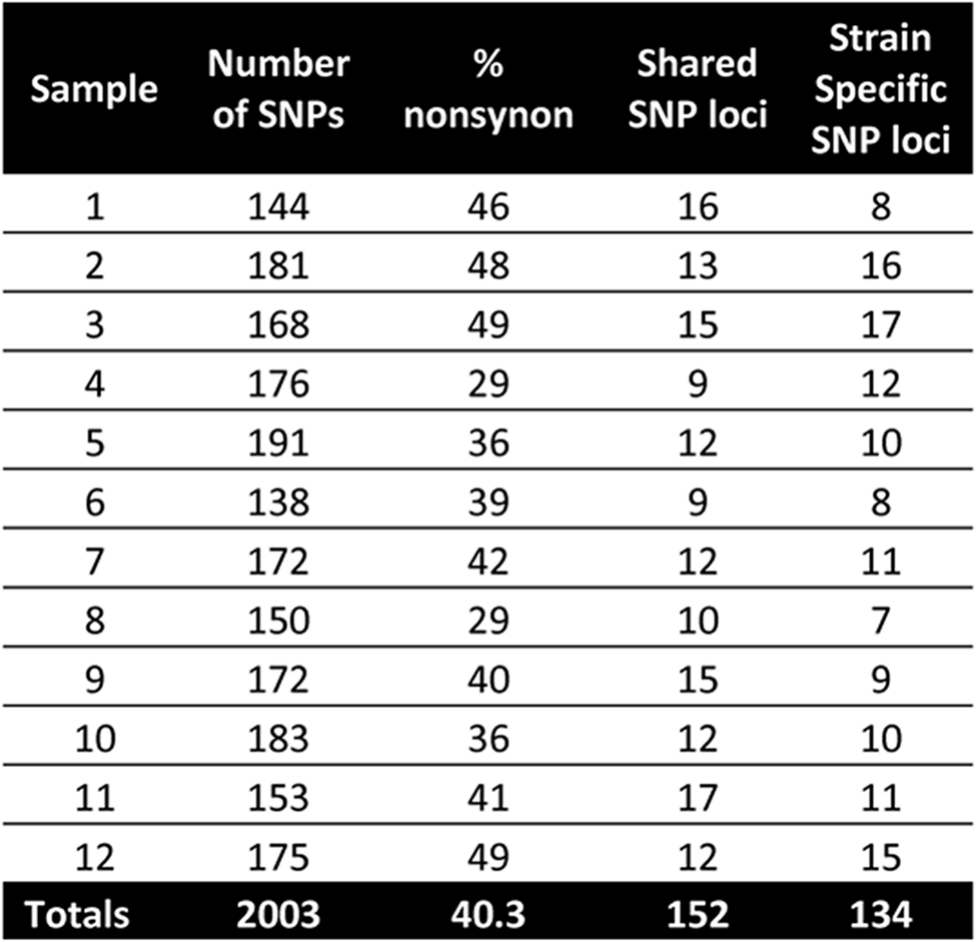
Annotation of the number of single nucleotide polymorphisms (SNP), percentage of SNPs which were nonsyonymous, number of shared SNP loci among study isolates (1 through 12) and number of strain specific SNPs among study isolates.

Optical mapping of six selected bacterial isolates identified a close similarity (<1% variation) to the USA300 reference strains. This was confirmed for all twelve isolates by NGS. MLST showed that all isolates were sequence type (ST) 8 and aligned strongly to staphylococcal cassette chromosome (SCC) mec type IVa. MVLST revealed differences between the isolates and both USA300 strains in two virulence loci (HlgA and sdrC) and differences among the isolates in the gamma hemolysin (HlgA) locus (Fig 1). There were no differences among isolates at the sdrC locus (the dendrogram is not shown). Optical mapping and NGS differentiated the isolates from the USA300 strains by identifying the presence and location of limited insertions and deletions in various specimens. There were no rearrangements or copy number variations identified between the samples and the USA300 strains. With optical mapping and NGS, 40 Kb insertions were detected in isolates 7 and 10. An additional 10 Kb deletion was detected in isolate 9. Isolates 2, 4, 8 were not distinguishable from the USA300 strains by optical mapping. NGS identified genetic heterogeneity among the study isolates when SNP analysis was performed with respect to the USA300 reference strains. Each isolate showed an average of 99.6 synonymous and 67.3 nonsynonymous SNPs. There was an average of 11 strain specific, non-synonymous SNPs per isolate (Fig 2).

Seventy-eight virulence genes of USA300were identified through literature review (Table 3) [12,13,15–19,24–31]. The rate of occurrence of these virulence genes was determined for the two reference strains and for our isolates. Fifty-nine and 65 of these genes were present in USA300 FPR3757 and TCH1516, respectively. There was a significantly higher representation of these genes in our clinical isolates when compared to the reference strains, with an average of 76 (median of 78) virulence genes in the study isolates (p = 0.0124).

**Table 3:** Virulence genes and SNP occurrence among study isolates and USA 300 MRSA reference strains.

				UTSW/CMC MRSA Isolates											
		USA 300 FPR3757	USA 300 TCH1516	Isolate #7	Isolate #1	Isolate #10	Isolate #4	Isolate #9	Isolate #6	Isoalte #12	Isolate #11	Isolate #3	Isolate #5	Isolate #8	Isolate #2
				Severity of Illness Score											
				0	3	3	5	6	7	7	8	8	8	9	9
Gene	Putative function	* *		Not Severe (NS)				Severe (S)							
agr	Global regulator	P	P	P	P	P	P	P	P	P	P	P	P	P	P
arsB	arsenic efflux pump protein	**A**	P	P	P	P	P	**SNP**	P	P	P	P	P	P	P
cap5A	capsular polysaccharide	P	P	P	P	P	P	P	P	P	P	P	P	P	P
cap5B	capsular polysaccharide	P	P	P	P	P	P	P	P	P	P	**SNP**	P	P	P
cap5C	capsular polysaccharide	P	P	P	P	P	P	P	P	P	P	P	P	P	P
cap5D	capsular polysaccharide	P	P	P	P	P	P	P	P	P	P	P	P	P	P
cap5E	capsular polysaccharide	P	P	P	P	P	P	P	P	P	P	P	P	P	P
cap5F	capsular polysaccharide	P	P	P	P	P	P	P	P	P	P	P	P	P	P
cap5G	capsular polysaccharide	P	**A**	P	P	P	P	P	P	P	P	P	P	P	P
cap5H	capsular polysaccharide	P	P	P	P	P	P	P	P	P	**SNP**	P	P	P	P
cap5I	capsular polysaccharide	P	P	P	P	P	P	P	P	P	P	P	P	P	P
cap5J	capsular polysaccharide	P	P	P	P	P	P	P	P	P	P	P	P	P	P
cap5K	capsular polysaccharide	P	P	P	P	P	P	P	**SNP**	P	P	P	P	P	P
cap5L	capsular polysaccharide	P	P	P	P	P	P	P	P	P	P	P	P	P	P
cap5M	capsular polysaccharide	P	P	P	P	P	P	P	P	P	P	P	P	P	P
cap5N	capsular polysaccharide	P	P	P	P	P	P	P	P	P	P	P	P	P	P
cap5O	capsular polysaccharide	P	P	P	P	P	P	P	P	P	P	P	P	P	P
ccra	cassette chromosome recombinase	P	P	P	P	P	P	P	P	P	P	P	P	P	P
clfA	Adhesin for fibrinogen	**A**	P	P	P	P	**SNP**	P	P	P	P	P	P	P	P
chp	chemotaxis-inhibiting protein CHIPS	**A**	P	P	P	P	P	P	P	P	P	P	P	P	P
coa	staphylocoagulase	P	**A**	P	P	P	P	P	P	P	P	P	P	**SNP**	P
cody	transcriptional repressor CodY	P	**A**	P	P	P	P	P	P	P	P	P	P	P	P
eap	extracellular adherence protein Eap	**A**	P	P	P	P	P	P	P	P	P	P	P	P	P
ear	protein coding	P	P	P	**SNP**	P	P	**A**	P	P	**A**	**A**	**A**	P	P
ebpS	Adhesin for elastin	P	P	P	P	P	P	P	P	P	P	P	P	P	P
epiB	lantibiotic epidermin biosynthesis protein	P	P	P	P	P	P	P	P	**SNP**	P	P	P	P	P
epiC	lantibiotic epidermin biosynthesis protein	P	P	P	P	P	P	P	P	P	P	P	P	P	P
efb	Binds to fibrinogen	P	**A**	P	P	P	P	P	P	P	P	P	P	P	P
essC	virulence protein EssC	**A**	P	P	P	P	P	P	P	P	P	P	P	P	P
fbpA	putative fibronectin/fibrinogen binding protein	**A**	P	P	P	P	P	P	P	P	P	P	P	P	P
fhuB	ferrichrome ABC transporter	**A**	P	P	P	P	P	P	P	P	P	P	P	P	P
fmt	fmt protein	P	P	P	P	P	P	P	P	P	P	P	P	P	P
fnbA	Adhesin for fibronectin	P	P	P	P	P	P	P	P	P	P	P	P	P	P
fnbB	Adhesin for fibronectin	P	P	P	P	P	P	P	P	P	P	P	P	P	P
fur	iron uptake regulatory protein	P	**A**	P	P	P	P	P	P	P	P	P	P	P	P
hlb	truncated beta-hemolysin MW2	**A**	P	P	P	P	P	P	P	P	P	P	P	P	P
hld	delta-hemolysin	**A**	P	P	P	P	P	P	P	P	P	P	P	P	P
hlgA	gamma hemolysin	P	P	P	P	P	P	P	P	P	P	P	P	P	P
hly	alpha-hemolysin precursor	**A**	P	P	P	P	P	P	P	P	P	P	P	P	P
hsdM	type I site-specific deoxyribonuclease	**A**	P	**A**	P	P	P	P	P	P	**A**	P	P	P	P
htrA	serine protease HtrA	P	**A**	P	P	P	P	P	P	P	P	P	P	P	P
hysA	hyaluronate lyase precursor	P	P	P	P	P	P	P	P	P	P	P	P	P	P
icaA	Polysaccharide intercellular adhesin	**A**	P	P	P	P	**SNP**	P	P	P	P	P	P	P	P
icaB	Polysaccharide intercellular adhesin	P	P	P	P	P	P	P	P	P	P	P	P	P	P
icaC	Polysaccharide intercellular adhesin	P	P	P	P	P	P	P	P	P	P	P	P	P	P
icaD	Polysaccharide intercellular adhesin	**A**	P	P	P	P	P	P	P	P	P	P	P	P	P
ksgA	dimethyladenosine transferase	P	P	P	P	P	P	P	P	P	P	P	P	P	P
ileS	isoleucyl-tRNA synthetase	P	P	P	P	P	P	P	P	P	P	**SNP**	P	P	P
IpI3	hypothetical protein	P	**A**	P	P	P	P	P	P	P	P	P	P	P	P
IpI7	hypothetical protein	**A**	P	P	P	P	P	P	P	P	P	P	P	P	P
IpI11	hypothetical protein	**A**	P	P	P	P	P	P	P	P	P	P	P	P	P
lukD	destruction of white blood cells	P	P	P	P	P	P	P	P	**SNP**	P	P	P	P	P
lukE	destruction of white blood cells	P	P	P	P	P	P	P	P	P	P	P	P	P	P
nuc	nuclease	P	P	P	P	P	P	P	P	**SNP**	P	P	P	P	P
plc	1-phosphatidylinositol phosphodiesterase	P	**A**	P	P	P	P	P	P	P	P	**SNP**	P	P	P
rot	repressor of toxins Rot	P	P	P	P	P	P	P	P	P	P	P	P	P	P
Rsbu	sigmaB regulation protein RsbU	P	P	P	P	P	P	P	P	P	P	P	P	P	P
SaeR	DNA-binding response regulator SaeR	P	P	P	P	P	P	P	P	P	P	P	P	P	P
sak	staphylokinase precursor	P	P	P	P	P	P	P	P	P	P	P	P	P	P
sarA	accessory regulator A	P	P	P	P	P	P	P	P	P	P	P	P	P	P
sarH1	staphylococcal accessory regulator	**A**	P	P	P	P	P	P	P	P	P	P	P	P	P
sarR	staphylococcal accessory regulator	**A**	P	P	P	P	P	P	P	P	P	P	P	P	P
sarT	staphylococcal accessory regulator	P	P	P	P	P	P	P	P	P	P	P	P	P	P
sarU	accessory regulator U	P	P	P	P	P	P	P	P	P	P	P	P	P	P
sarX	hypothetical protein	P	P	P	P	P	P	P	P	P	P	P	P	P	P
sbi	IgG binding protein	P	**A**	P	P	P	P	**SNP**	P	P	P	P	P	P	P
scrA	sucrose-specific PTS tranporter protein	**A**	P	P	P	P	P	P	P	P	P	P	P	P	P
sek	enterotoxin	P	**A**	P	P	P	P	**A**	P	P	**A**	**A**	**A**	P	P
seq	enterotoxin Q	P	**A**	P	P	P	P	**A**	P	P	**A**	P	**A**	P	P
set7	Unknown	P	**A**	P	P	P	P	P	P	P	P	P	P	P	P
spa	Binds Fc domain of immunoglobulin	**A**	P	P	P	P	P	P	P	P	P	P	P	P	P
splA	serine protease SplA	P	P	P	P	P	P	P	P	P	P	P	P	P	**SNP**
splB	serine protease SplB	P	P	P	P	P	P	P	P	P	P	P	P	P	P
splC	serine protease SplC	P	P	P	P	P	P	P	P	P	P	P	P	P	P
splD	serine protease SplD	P	P	P	P	P	P	P	P	P	P	P	P	P	P
splF	serine protease SplF	P	P	P	P	P	P	P	P	P	P	P	P	P	P
sspB	cysteine protease precursor	P	**A**	P	P	P	P	P	P	**SNP**	P	P	P	P	P
tcaB	teicoplanin resistance	P	P	P	P	P	P	P	P	P	P	P	P	P	P

University of Texas Southwestern (UTSW); Children’s Medical Center of Dallas (CMC); Methicillin-resistant *Staphylococcus aureus* (MRSA); Present (P); Absent (A); Single Nucleotide Polymorphism (SNP).

Univariate analysis of the MRSA isolates did not identify any individual virulence gene or SNP which was significantly associated with low severity (score ≤ 5) versus high severity of illness (score > 5) of the affected children. One combination of genes (sek and ear) was noted to be absent in 4 out of 8 strains which caused infection in children with severe illness, but present in all 4 children who did not have severe illness. This association was not statistically significant (p = 0.208), but may be biologically significant.

University of Texas Southwestern (UTSW); Children’s Medical Center of Dallas (CMC); Methicillin-resistant *Staphylococcus aureus* (MRSA); Present (P); Absent (A); Single Nucleotide Polymorphism (SNP).

### Phylogenetic Analysis

**Fig 3:**
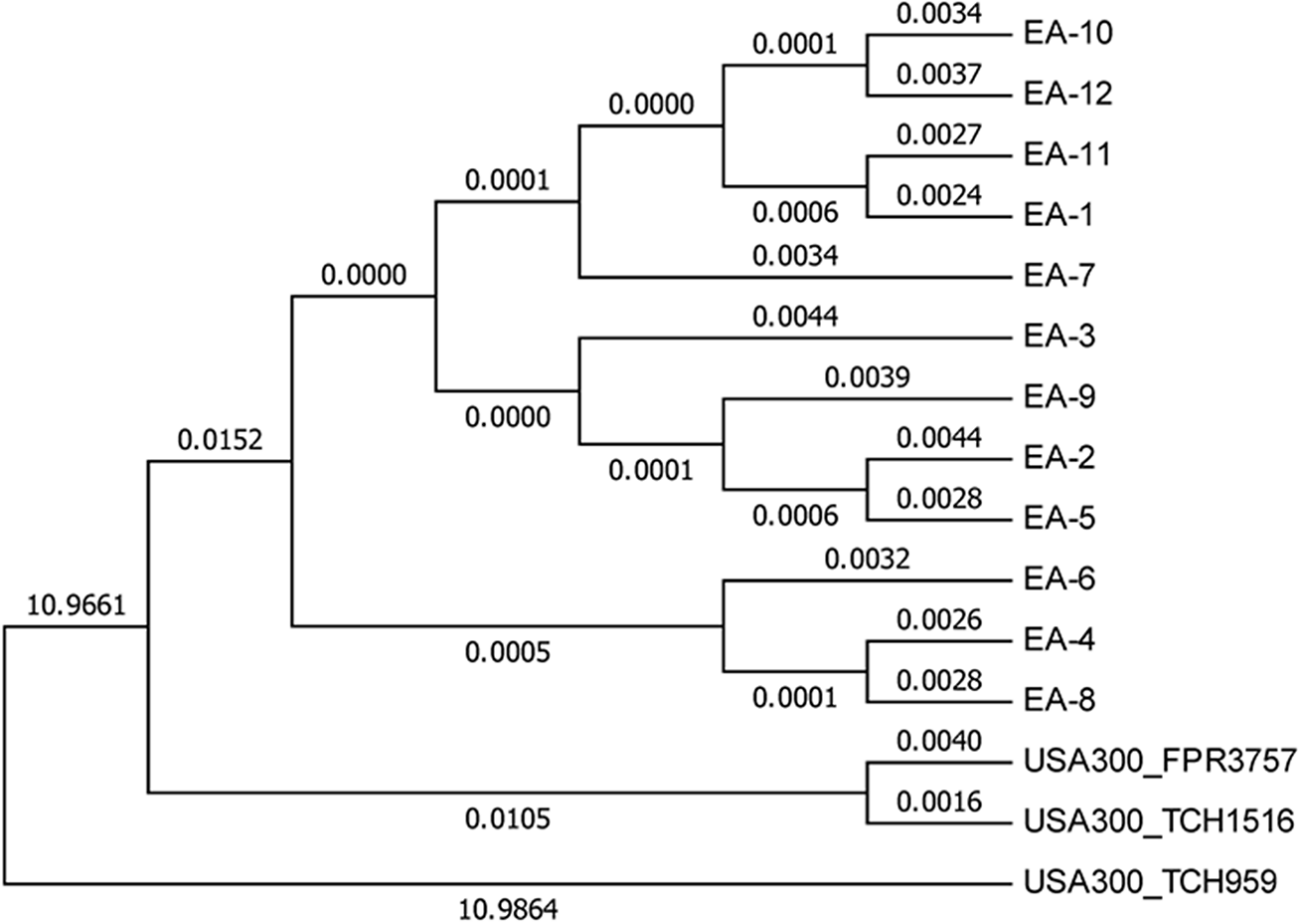
Dendrogram of the phylogenetic analysis by maximum likelihood (PAML) comparing study isolates (EA-1 through EA-12) with reference strains (FPR3757; TCH1516; and TCH959). The evolutionary distances between sequences is indicated by computing the proportion of nucleotide differences between each pair of sequences.

There was evolutionary distance identified between each of the study isolates. Evolutionary distance was also identified between the aggregate of study isolates and the USA 300 MRSA reference strains (FPR3757 and TCH1516) (Fig 3). The greatest evolutionary distance was noted between the superficial skin and soft tissue MSSA reference strain (TCH959) and the remaining isolates, including both MRSA reference strains and all study isolates.

The pathogenetic mechanisms underlying the variation of clinical manifestations of children with osteomyelitis due to MRSA are incompletely understood. We have previously reported that children with invasive MRSA infections display an over-expression of innate immunity and under-expression of adaptive immunity and that specific genes correlate with severity of illness [32]. Although host factors are likely to play a role in clinical variability, attention must still be directed to the bacteria in light of the diverse virulence capabilities of MRSA. There is increasing evidence to suggest that a cascade of events may permit *Staphylococcus aureus* to survive in the bloodstream, avoid destruction by neutrophil phagocytosis, traverse the endothelium to gain access to deep structures, and adhere to collagen in bone so as to permit local skeletal invasion and destruction [24,25,27,29,33]. These events correspond to up-regulation and expression of specific bacterial genes related to these activities [34,35]. It is therefore likely that genetic loci variations might act to amplify or attenuate the severity of infection during a complex cascade of events.

In this study, we have demonstrated bacterial genomic heterogeneity among MRSA isolates from children with variable clinical manifestations of disease. This bacterial heterogeneity was detected by next generation technologies and would not have been identified using standard methods of PFGE, or MLST. All study isolates were Sequence Type 8 USA 300 MRSA (SCC mec type IVa). However, there was variability of our isolates compared to standard USA 300 reference strains when analyzed by MVLST, optical mapping and NGS. MVLST demonstrated heterogeneity of the gamma hemolysin gene of isolates at the HlgA locus. Such a difference may prove to be important in permitting varying degrees of bacterial survival within the bloodstream so as to enhance dissemination of disease. Without hemolytic capability, *S*. *aureus* would be unable to survive in human blood. The up-regulation of iron metabolic pathways of the organism is required because the concentration of free iron is 10^−11^ times lower than that necessary for bacterial survival [33]. While several other virulence determinants of *S*. *aureus* demonstrate genetic redundancy due to the presence of alternative subunits, the hemolysin subunits do not have this feature [33]. Gamma-hemolysin is a bicomponent pore-forming leukotoxin encoded by three genes, hlgA, hlgB, and hlgC, all within the same operon. A recent study of genetic evolution of colonizing strains of MRSA found a minor rearrangement and accumulation of synonymous and nonsynonymous SNPs in clusters of hlgA and hlgC genes [36]. The polymorphisms of the gamma hemolysin subunit identified in our study may play a role in varying hemolytic capability of the organisms. We found that only 25% of children with mild severity of illness (scores of 5 or less) had positive blood cultures, whereas 100% of children with scores greater than 5 had bacteremia.

A critical barrier to progress in this field has been the inability to prove that specific bacterial virulence determinants are responsible for clinical manifestations of infection. Some evidence suggests an association between bacterial strains which express Panton Valentine leukocidin (PVL) and severe complications, such as deep venous thrombosis and septic pulmonary emboli [5,7,27]. However, there is limited direct evidence of the role of single virulence determinants in the pathogenesis of virulent strains [37]. The redundancy of bacterial genes coding for leukocyte defense and the presence of PVL in non-virulent strains imply a more complex cascade of events than that which is explained by the activity of an isolated virulence gene [34,37]. In support of this, one group of investigators evaluated 33 putative virulence factors in bacterial isolates from individuals with either invasive or non-invasive *S*. *aureus* infections and identified a combination of virulence genes (*fnbA*, *cna*, *sdrE*, *sej*, *eta*, *hlg*, and *ica*) that were significantly associated with invasive disease [25]. No single factor predominated and the effects of the virulence genes appeared to be cumulative [25]. However, the study was done using PCR technology focusing on a limited number of virulence genes and it did not assess the more expansive genome of *S*. *aureus*. Recent evidence suggests that the difference in pathogenesis between strains is more likely due to subtle changes rather than to large-scale acquisition of virulence genes [38]. One study found that bacterial heterogeneity can be discovered in closely related bacterial isolates by investigating SNPs [38]. The investigators sequenced ten MRSA USA300 strains from eight different states and found that 80% of the isolates had very few SNPs and appeared closely related [38].

An important finding of this study is the heterogeneity of the bacterial isolates at the SNP level within a single community. The disproportionate occurrence of SNPs in virulence genes among children who demonstrated severe illness in our study requires further investigation to see if any pattern of SNP variation may lead to amplification or attenuation of the pathogenic cascade. It is possible that SNPs could represent variable and transient events within a bacterial species. However, recent literature suggests that genomic differences between closely related bacterial strains are indeed not representative of spontaneous mutations because they have already undergone selection [39]. The inter-isolate SNP variation identified in this study therefore most likely corresponds to an established heterogeneity of the isolates.

An important limitation of this study is the small sample size. This was intentionally designed as an exploratory study with a narrow focus on children within a single community who were infected with a single bacterial isolate (MRSA) within a limited timeframe. Because of the extremely small sample size, there is limited power to detect significant differences of clinical severity due to selected virulence genes, either in isolation or combination. However, our findings suggest that the greater discriminatory power of NGS may succeed in delineating this information when applied to a substantially larger sample size using appropriate controls, including methicillin sensitive strains, isolates from superficial skin and soft tissue infections, and nasal swab isolates from asymptomatic hosts. A second limitation of this study is the lack of information regarding the gene expression of the isolates. Ultimately, it will be necessary to determine the transcriptome of the isolates at a variety of stages during disease pathogenesis. It is possible that nonsynonymous SNPs could result in amplification or attenuation of the cascade of events during the host-pathogen interaction due to altered bacterial proteomics. One other limitation of our study pertains to the selection of reference strains. Within GenBank, there are currently no reference strains with complete genomes that have been isolated from individuals with similar clinical conditions to the children with acute hematogenous osteomyelitis within our community. An isolate from an HIV positive adult residing in California who had a superficial skin and soft tissue infection is clinically dissimilar to that found among population of children with deep infection in Dallas. This is also true regarding the isolate from the adolescent with “severe sepsis syndrome” in Houston in 2007 due the chronological and geographic separation of cases. This limitation points to the need for the submission of new whole genome sequences of new strains to GenBank with relevance to the clinical phenomena being studied.

## Conclusions

This exploratory study represents the most extensive effort to date utilizing NGS and molecular epidemiology to identify bacterial genomic heterogeneity among MRSA isolates affecting a well-defined population of children who demonstrated diverse severity of illness. NGS offers sufficient discriminatory power to gradually reveal the underlying pathogenetic mechanisms of acute osteomyelitis in children when carefully considered in context of the disease manifestations. Although causation cannot be derived from our findings, this study offers direction for further investigation including studies of bacterial gene expression and applied cell biology including hemolysis, neutrophil killing, and endothelial cell uptake when exposed to bacteria with genetic differences. The intention of this study was to assess the feasibility of continuing this line of inquiry, using higher statistical power through a much larger sample size. We conclude that next generation sequencing technology confirmed the genomic variation among our isolates. We are unable to establish a relationship of this bacterial heterogeneity and the variation in host response due to the small sample size. However, there is enough support to encourage further investigation with a carefully designed and appropriately powered study. Ultimately, the ability to identify the virulence potential of individual clinical isolates early in the course of illness would impact clinical care and allow devotion of appropriate resources to children with the greatest need. For example, rapid genetic testing of bacteria identified in the initial blood culture obtained in the emergency room from a child infected by a highly virulent strain might lead to urgent acquisition of magnetic resonance imaging and surgical decompression of the foci of infection. It also might prompt investigation for deep venous thrombosis which would be addressed with appropriate anticoagulation therapy or IVC filter placement. The admitting team might choose intensive care monitoring to allow for pulmonary support in anticipation of respiratory compromise after early recognition of septic pulmonary emboli, identified by CT scan, which otherwise might not have been obtained.
